# The genetic basis of the fitness costs of antimicrobial resistance: a meta-analysis approach

**DOI:** 10.1111/eva.12202

**Published:** 2014-12-12

**Authors:** Tom Vogwill, R Craig MacLean

**Affiliations:** Department of Zoology, University of OxfordOxford, UK

**Keywords:** adaptation, antibiotic resistance, antimicrobial resistance, fitness cost, microbes, mutation, plasmid

## Abstract

The evolution of antibiotic resistance carries a fitness cost, expressed in terms of reduced competitive ability in the absence of antibiotics. This cost plays a key role in the dynamics of resistance by generating selection against resistance when bacteria encounter an antibiotic-free environment. Previous work has shown that the cost of resistance is highly variable, but the underlying causes remain poorly understood. Here, we use a meta-analysis of the published resistance literature to determine how the genetic basis of resistance influences its cost. We find that on average chromosomal resistance mutations carry a larger cost than acquiring resistance via a plasmid. This may explain why resistance often evolves by plasmid acquisition. Second, we find that the cost of plasmid acquisition increases with the breadth of its resistance range. This suggests a potentially important limit on the evolution of extensive multidrug resistance via plasmids. We also find that epistasis can significantly alter the cost of mutational resistance. Overall, our study shows that the cost of antimicrobial resistance can be partially explained by its genetic basis. It also highlights both the danger associated with plasmidborne resistance and the need to understand why resistance plasmids carry a relatively low cost.

## Introduction

Antimicrobial resistance carries a fitness cost that is expressed in terms of reduced growth rate, competitive ability or virulence (reviewed in Andersson 2006; Andersson and Hughes 2010). This cost of resistance is predicted to play a key role in the evolutionary dynamics of resistance because it generates selection against resistance (e.g. Austin et al. 1997, 1999; Lipsitch et al. 2000; zur Wiesch et al. 2011). This is particularly important when bacteria encounter an antibiotic-free environment, as occurs when patients stop using an antibiotic or during transmission between hosts. Because of this central role, the costs of resistance have now been measured in well over 100 studies spanning a wide diversity of resistance mechanisms and pathogens. These studies have found that the cost of resistance is highly variable. For example, some studies have reported costs of resistance of >50% (e.g. Binet and Maurelli 2005; Norström et al. 2007; Pränting and Andersson 2011), while other studies have found that resistance carries little if any cost (e.g. Pränting et al. 2008; Sandegren et al. 2008; Castaneda-Garcia et al. 2009). It is perhaps not surprising that the cost of resistance is highly variable, as it is influenced by a wide variety of factors. These include the biochemical effects of specific resistance mutations (e.g. Andersson et al. 1986; Schrag and Perrot 1996; Macvanin et al. 2000; Zorzet et al. 2010), as well as the ecological and genetic background in which the cost of resistance is measured (e.g. Paulander et al. 2009; Trindade et al. 2009; Ward et al. 2009; Hall and MacLean 2011). Because of this complexity, it is unlikely that we will ever be able to fully explain the cost of resistance.

However, one factor that could potentially play a key role in the cost of resistance is the underlying genetic mechanism of resistance. At the broadest level, resistance can evolve either as a result of chromosomal mutation or via the acquisition of a mobile genetic element (MGE) (Levy and Marshall 2004; Alekshun and Levy 2007). Intuition suggests that the cost of resistance is likely to differ between MGEs and mutations (MacLean et al. 2010). For example, in addition to resistance genes, MGEs impose an additional burden on their hosts because they not only carry genes for MGE transmission but also encode functions unrelated to antibiotic resistance (reviewed in Rankin et al. 2011). More generally, there are potential conflicts of interests between MGEs and their host bacteria, whereby maximizing MGE fitness does not maximize host fitness (Mc Ginty and Rankin 2012). For example, trade-offs can exists between the vertical and horizontal transmission of MGEs (Mc Ginty and Rankin 2012), or as a result of kin selection for cooperation among MGEs (Nogueira et al. 2009; Mc Ginty et al. 2011). Conversely, it could be argued that many resistance mutations are likely to have a greater cost than MGEs, as they often modify essential genes which are otherwise highly conserved (Alekshun and Levy 2007).

Importantly, there is also a considerable genetic diversity of resistance mechanisms within each of these broad classes. For example, mutational resistance can evolve via changes to a wide variety of genes (reviewed in Alekshun and Levy 2007). These include mutations in highly conserved proteins that play key roles in cellular physiology, such as ribosomal proteins or RNA polymerase. However, resistance can also evolve via mutations in accessory genes that seem to be primarily involved in antibiotic resistance, such as β-lactamase enzymes, or genes involved in broad variety of cellular processes, such as efflux pumps. It has been shown that the biochemical effects of alternative resistance mutations in the same gene can explain why some resistance *mutations* are more costly than others (e.g. Andersson et al. 1986; Schrag and Perrot 1996; Macvanin et al. 2000; Zorzet et al. 2010). However, it is still unclear if certain genetic mechanisms of resistance are consistently more costly than others. Similarly, intuition suggests that the genetic diversity that exists within MGEs may influence the cost of resistance. For example, at one extreme, there are small plasmids (<10 kb) that may only carry a single resistance determinant and little else besides the genes involved in plasmid replication (e.g. San Millan et al. 2009). In contrast, large plasmids (>100 kb) can carry in excess of 10 resistance determinant as well as a wide variety of genes involved in other traits (e.g. Sandegren et al. 2012). It is clear that the underlying mechanisms that generate a cost of MGE acquisition are complex and diverse (Baltrus 2013). However, it is unclear how this diversity may impact the cost of resistance.

The majority of papers examining costs of resistance contain a relatively small number of estimates of the cost of resistance, although this ranges from one unique mutation to more than 60, with a mean of 10 isolates per study. Usually, this is the cost of a single specific mechanism which provides resistance to only a single family of antibiotics, or often a small number of MGEs which provide resistance to one or more antibiotic families. Therefore, to infer the relative cost of different mechanisms of resistance in is necessary to compare across articles. We therefore performed a quantitative meta-analysis of published estimates of the fitness cost of resistance. Specifically, we calculated the mean cost of resistance for many independent papers and then analysed this data in relation to the various mechanisms of resistance. Our analysis initially focuses on the methodology of measuring fitness costs and whether different types of assays give comparable outcomes. We then question whether the genetic basis of resistance determines its cost by comparing the cost of MGEs and chromosomal mutations. MGEs are highly diverse, but almost all of the estimates of the cost of acquiring MGEs come from plasmids. We therefore focus our analysis on the cost of evolving resistance by plasmid acquisition versus the cost of via chromosomal mutation. We then examine which factors contribute to the cost of plasmid-mediated resistance, testing whether resistance range (i.e. the degree of multidrug resistance), plasmid size or host genetic background contributes to the cost of plasmid acquisition. We then examine chromosomal resistance mutations and test whether the molecular mechanism or the biochemical basis effect the cost of resistance. Finally, we analyse papers which studied multistep resistance evolution and test for the role of epistasis in resistance evolution.

## Materials and methods

### Literature search

Papers reporting the fitness costs of newly acquired antibiotic resistance were initially collected by searching in Pubmed, Web of Knowledge and Google scholar using the search terms ‘antibiotic resistance or antimicrobial resistance’ and ‘cost,’ followed by the use of secondary filters (fitness/biological/physiological). Search results were limited to papers which were at least available online by the 31st December 2012. No start date was specified. The search was limited to English language publications. The search was limited to peer-reviewed articles. Additional searches were also performed to increase the number of papers reporting the fitness of mobile genetic elements (MGE), using the terms ‘fitness’ and ‘plasmid,’ ‘transposon,’ ‘integron’ or ‘genomic island’. We also conducted manual searches of the bibliographies of each paper which met our inclusion criteria.

### Criteria for inclusion

**Figure 1:**
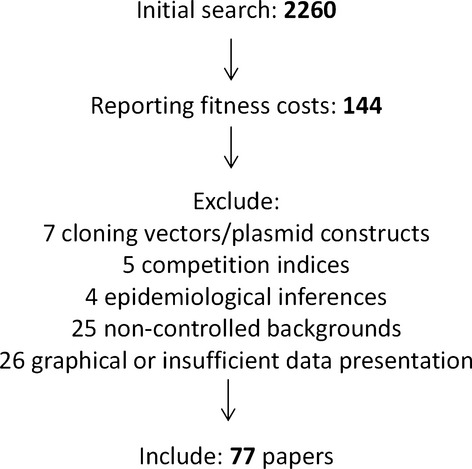
Overview of the inclusion process.

To be included, papers had to report the results of fitness assays performed on antimicrobial resistant bacteria and had to report the findings *numerically*. This could be of either the fitness of individual resistant mutants or the mean fitness of many mutants. Fitness measures had to be relative to a control strain which only differs in the presence of the cause of resistance [either a chromosomal mutation (SNP, knockout, etc) or acquisition of a MGE]. We therefore excluded papers which compared clinically isolated mutants which were either susceptible or resistance, as these isolates can differ in more than just the mechanism of resistance. We chose to analyse the data in terms of fitness or *w,* where by definition the susceptible ancestor was a fitness of 1, with costly mutations having scores of <1 and beneficial mutations having a score in excess of 1. If the data were reported in an unstandardized form (i.e. as raw growth rate or doublings per hour not relative to a susceptible ancestral strain), manual standardization was performed by dividing by the relevant susceptible ancestor or control, if reported. If a control value was not reported, this study was excluded. If the data were reported in terms of selective coefficients (*s*)*,* this was transformed into our working measure of *w*. Papers using competition indexes, the ratio of resistant to susceptible bacteria at the end of a set period of time, were excluded because this measure does not include any aspect of generation/doubling time and consequently is not comparable outside of the exact context in which it is measured. We also excluded any mobile elements which had been engineered or altered by laboratory work, therefore limiting our analysis to wild-type resistance plasmids. A flowchart of our inclusion process is presented in Fig.1.

### Rationale

To avoid biasing our results in favour of whichever paper measured the highest number of resistant isolates, for the majority of the analysis, we calculate means for each paper and therefore each published study is represented by a single data point. This is a standard procedure when performing a meta-analysis as the unit of replication is the individual study. This is chiefly to avoid confounding different levels of replication (here it would be confounding variation between resistance mutations with variation between resistance mechanisms). Additionally, this approach prevents the analysis from becoming essentially an analysis of certain mechanisms of resistance which are highly experimentally tractable. Not only have these mechanisms been studied by more papers, but more mutations per paper when done so. For example, rifampicin-resistant mutations represent over 25% of individual mutations in our data set, but only 13% of papers studied rifampicin. Therefore, performing the analysis at the level of the mechanism is more reflective of the diversity of mechanisms of resistance. However, if a paper had measured resistance to more than one antibiotic family, these were included as separate data points. Similarly, if a paper had measured fitness costs for multiple different plasmids, these were also treated as separate data points. Finally, when testing if different methods provide correlated estimates of fitness for the same resistant isolate, for obvious reasons, we treat each resistant isolate as a different data point. If the same plasmid had been assayed by multiple papers, an average was taken from all available measurements. This only applied to one plasmid (*Escherichia coli* plasmid R1, two papers). However, none of the outcomes of statistical tests were affected if each of these reports were included as separate data points, or if either of the two reports were solely included.

### Data analysis

Most fitness costs of antimicrobial resistance are presented as the ratio of the fitness of resistant mutants to the fitness of its susceptible ancestor. The meta-analysis of so-called response-ratios is well established (Hedges et al. 1999). However, to correctly calculate the weightings essential for a ‘formal’ meta-analysis, it is necessary to know the underlying means, standard deviations and sample sizes for the two values used to calculate the ratio. These are almost never reported for fitness costs, particularly for competition experiments that are considered the gold-standard for measuring the fitness of microorganisms. Although other methods for weighting studies do exist, given the small sample size of most studies reporting fitness costs (mean of 10 isolates, median of four, mode of one isolate), these methods would not result in accurate weightings (generally *n* = 5 is required for most weighting procedures) (Hedges et al. 1999). We therefore perform an unweighted analysis of published fitness costs using conventional statistics such as anova, *t*-tests, Pearson's correlations, etc. Reviews of meta-analysis suggest this approach when published data are missing information required for formal meta-analysis (Gurevitch and Hedges 1999; Gurevitch et al. 2001). However, these reviews also caution that if some papers have far greater sample sizes or sample variances than others, the results of unweighted analyses can be inaccurate or possibly meaningless. However, we would argue that this concern is less applicable when analysing laboratory measurements of microbial fitness. Although undoubtedly there is some study variance in the degree accuracy of these measurements, it is highly unlikely to be to the same extent as encountered with medical or ecological meta-analyses.

## Results

### Measuring the cost of resistance

**Figure 2:**
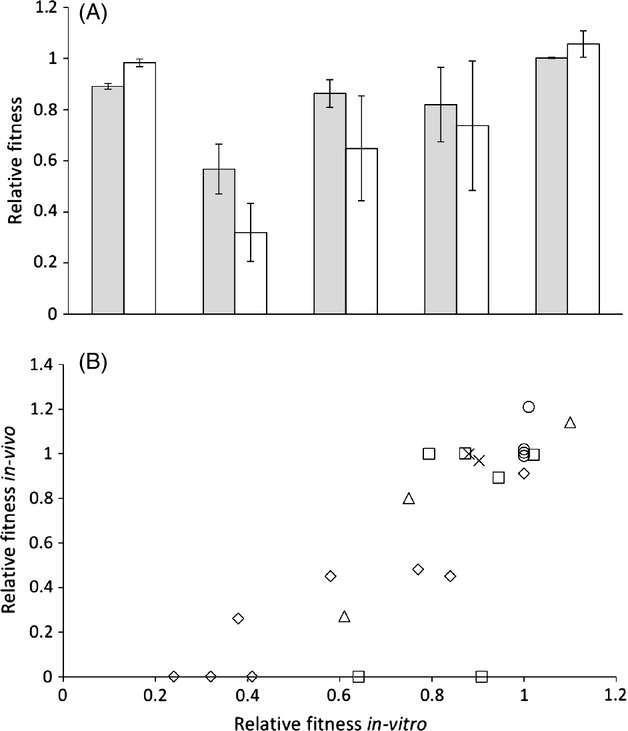
(A): There is no significant difference between the cost of antimicrobial resistance measured *in-vitro* (grey bars, mean fitness ± SEM) or in a mouse (white bars, mean fitness ± SEM). Each pair of bars represents a separate published paper. 2(B): Fitness in a mouse correlates with fitness *in-vitro*. Each set of symbols represents a different paper, with each point an independent mutation.

**Figure 3:**
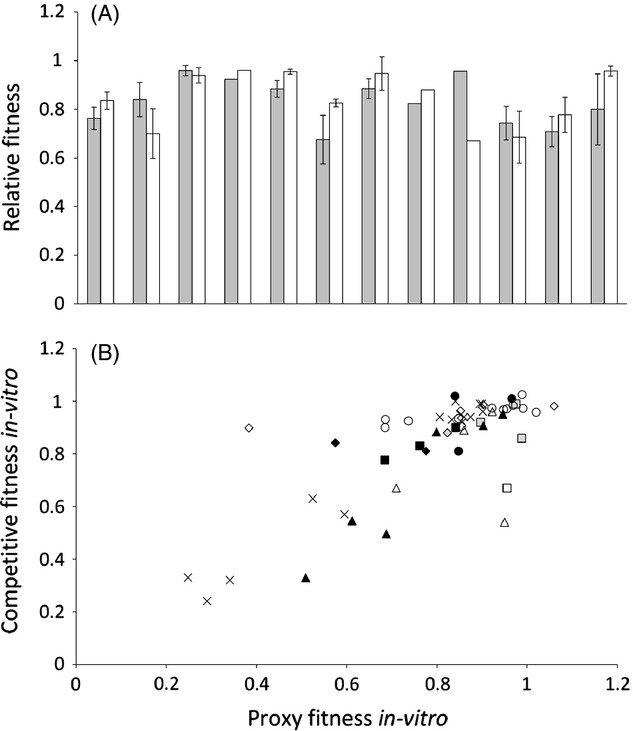
(A): There is no significant difference between the cost of resistance measured by a proxy (such as growth rate, density at a set time, etc.) (grey bars, mean ± SEM) or by direct competition assays (white bars, mean ± SEM). Each pair of bars is a separate published paper. 3(B): Competitive fitness correlates with growth rate. Each set of symbols represents a different paper, with each point an independent mutation.

After employing our criteria for inclusion, our search yielded 77 papers reporting the fitness cost of newly acquired antimicrobial resistance (Appendix S1), which represents a total of 822 independent resistant mutants. These papers used one or more of three broad methodologies to assess the fitness costs of resistance. Firstly, there are direct competition experiments against an ancestral strain performed *in vitro* (455 isolates). Secondly, there are *in vitro* proxy measures of fitness such as growth rates, doubling times, maximum yields, etc. (367 isolates). These measures are then standardized against the ancestral strain and used to infer the outcome of direct competitive interactions. Thirdly, there are competition experiments performed *in vivo,* or strictly speaking inside a mouse (23 isolates). Conveniently, several manuscripts have multiple methods on the same isolate, which thereby allows a comparison of the various methods. Specifically, 23 isolates (five papers) by both an *in vitro* and *in vivo* method, and 55 isolates (12 papers) had been assayed by both *in vitro* methods. If we use each paper as an independent data point, there is no significant difference in the mean cost of resistance between the *in vitro* and *in murine* fitness assays (Fig.2A; paired *t*-test on mean cost per manuscript, *in vitro* versus *in murine, t* = 1.17, df = 4, *P* = 0.307), nor between the two types of *in-vitro* assays (Fig.3A; paired *t*-test on mean cost per manuscript, growth rate versus competition, *t* = −0.394, df = 11, *P* = 0.703). This suggests that if a particular resistance mechanism is found to be either high or low cost by one assay, it is likely to be found to have the same relative cost by another methodology. Similarly, there are also significant correlations in fitness for individual resistance isolates which have been assayed in more than one way (Fig.2B: *in vitro* vs *In murine,* df = 21, *r* = 0.814, *P* < 0.001; Fig.3B: competition vs growth rate*,* df = 53, *r* = 0.763, *P* < 0.001). Both of these correlations remain significant if we control for the differences in the means of different papers (partial correlation between *in vitro* vs *in murine* controlling for differences between manuscripts, df = 20, *r* = 0.775, *P* < 0.001; partial correlation between competition versus growth rate controlling for differences between manuscripts, df = 52, *r* = 0.752, *P* < 0.001).

Due to the small sample size of *in vivo* measurements of the cost of resistance, we henceforth analyse only the *in vitro* measurements of the cost of resistance. We treat both proxy and direct competitive assays as equivalent measures of fitness. Therefore, for isolates assayed with both *in-vitro* assays, we calculate the average for each isolate prior to calculating the average for that study. This is necessary due to many manuscripts only performed both types of assays on a subset of isolates.

### Plasmid-mediated resistance carries a small cost

In general, antimicrobial resistance can either evolve by chromosomal mutation or by the acquisition of a mobile genetic element which carries one or more resistance gene. Most estimates of the cost of resistance come from studies that have measured the cost associated with chromosomal resistance mutations (60 studies, 78 antibiotic by study combinations, 760 total mutants). Although a broad diversity of MGEs can carry resistance genes, plasmids are by far the best characterized vectors of horizontal resistance transmission. Indeed, we found a large number of estimates of the cost of carrying plasmids containing resistance genes (49 plasmids from 16 studies), whereas we could only find costs for seven examples (from 4 papers) of other types of mobile elements which matched our criteria. We therefore decided to focus the analysis of the cost of MGEs exclusively on plasmids.

**Figure 4:**
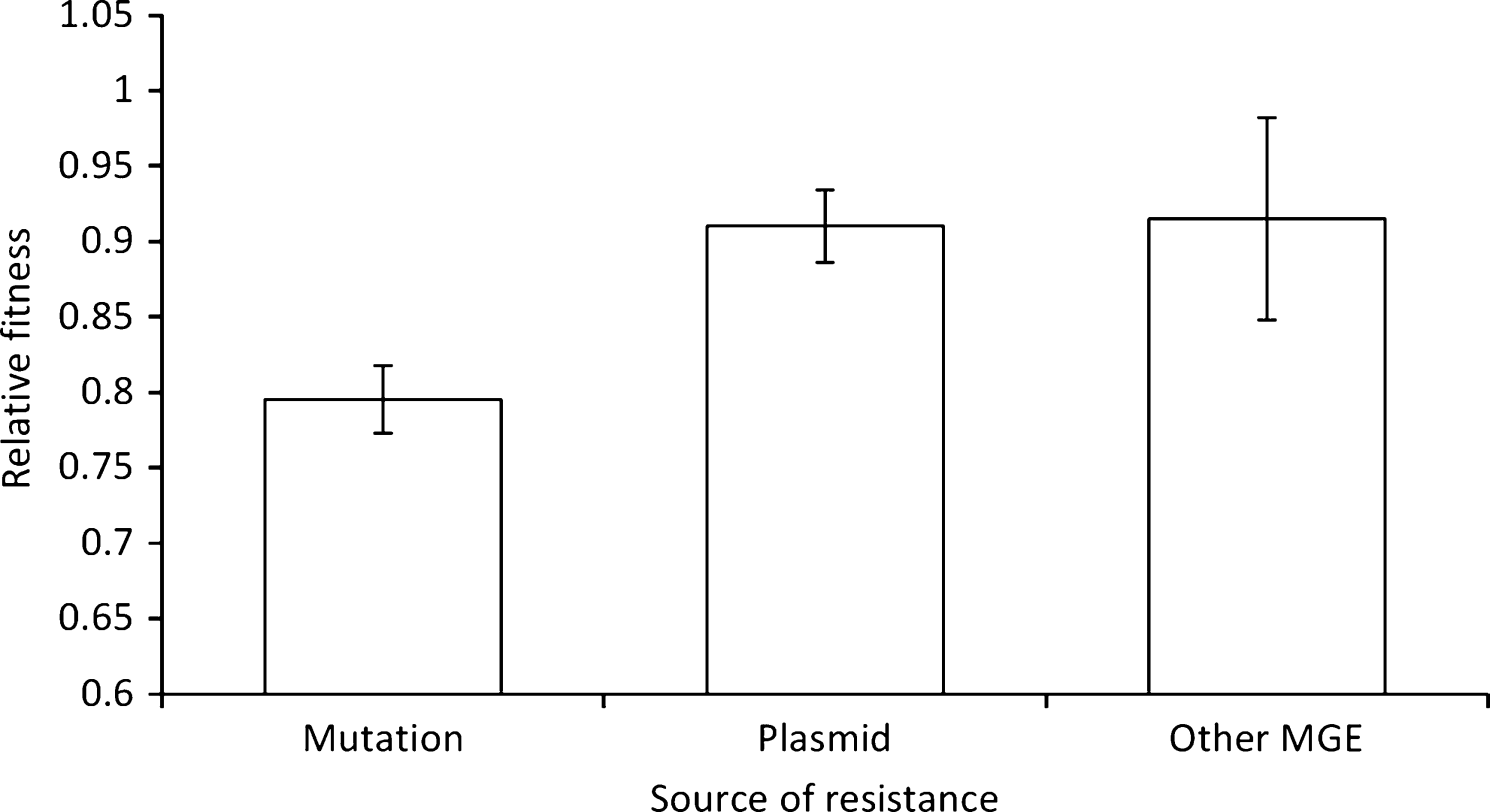
Evolving resistance by acquiring a plasmid has a smaller fitness cost than evolving resistance by mutation (bars show mean fitness ± SEM). The bars are the means of 78 mechanisms of mutational resistance, 49 plasmids, and 7 other mobile genetic elements, respectively.

To determine whether the genetic basis of resistance influences its cost, we compared the cost of resistance mutations and plasmids carrying resistance genes (Fig.4). The average cost of acquiring resistance via a plasmid (0.91 ± 0.024) is lower than the cost of resistance due to chromosomal mutations (0.79 ± 0.024). This difference is statistically significant when treating each plasmid as an independent observation (Fig.4; one-way anova, *F*_1,125_ = 12.421, *P* < 0.005) or, more conservatively, by treating each study as an independent observation (one-way anova, *F*_1,92_ = 5.14, *P* < 0.05). However, it could be argued that this is an inaccurate comparison, as the majority of plasmids carry resistance to multiple different antimicrobials. In contrast, the majority of mutational resistance mechanisms only result in high-level resistance to one family of antimicrobials. There are notable exceptions to this such as mutations effecting permeability (i.e. efflux pumps and porins), as well as mutations effecting global metabolism such as mutations leading to small colony variants. However, mutations which could directly result in multidrug resistance only represent 13.1% of our sample (10/78 chromosomal mechanisms). In contrast, MDR-plasmids represent 90% of the plasmids (43/49) in our sample, and on average, these plasmids confer resistance to 3.7 different antimicrobials. Therefore, if we perform a more appropriate comparison and only analyse mono-resistant mutations and plasmids, we still find a significant difference between the two genetic sources of resistance (*F*_1,72_ = 7.15, *P* < 0.01). The mean fitness for mutational resistance remains essentially unchanged (0.805 ± 0.022), whereas mono-resistant plasmids has now increased to 1.02 (± 0.112).

### The fitness costs of plasmid acquisition

**Figure 5:**
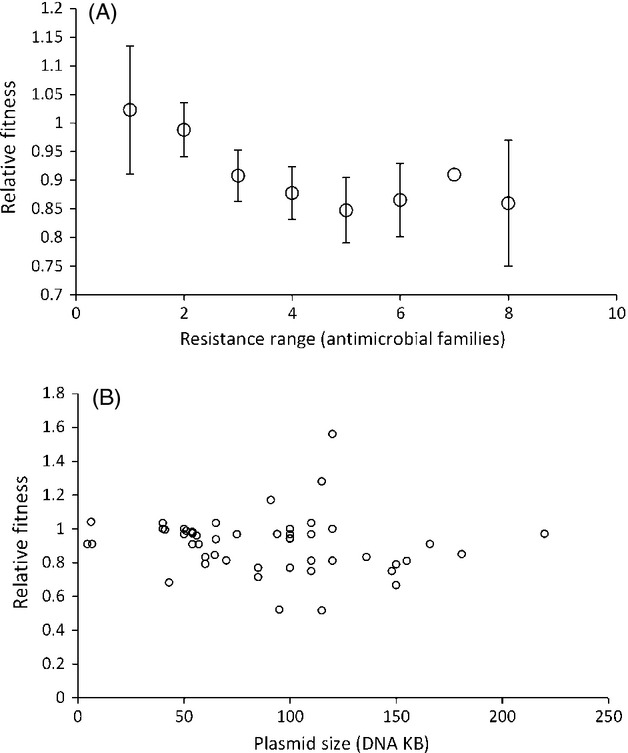
(A): The fitness cost of acquiring a plasmid increases in proportion to the resistance range of a plasmid. The resistance range of a plasmid is defined as the number of antimicrobial families to which phenotypic resistance is gained upon acquiring the plasmid, as reported in the relevant paper. Symbols represent mean fitness (± SEM), with a sample size of 6, 6, 14, 7, 8, 5, 1, and 2 plasmids, from left to right, respectively. 5(B): The size of a plasmid (DNA KB) does not significantly correlate with its fitness cost. Each point represents the published fitness cost of acquiring a resistance plasmid.

As the cost of mono-resistant plasmids is lower than the cost of all plasmids, it could be predicted that the cost of a plasmid should increase with increasing levels of multidrug resistance. Indeed, there is a significant correlation between the cost of a plasmid and the number of antimicrobial families to which that plasmid confers phenotypic resistance (Fig.5A; df = 47, *r* = −0.285 *P* < 0.05). This is not an artefact of larger plasmids carrying more resistances and thereby a greater number of other costly traits not associated with resistance, although larger plasmids do on average possess a greater range of resistances (df = 46, *r* = 0.428, *P* < 0.005). Specifically, plasmid size does not correlate with its fitness cost (Fig.5B; df = 46, *r* = −0.127, *P* = 0.390), nor does adding plasmid size improve the strength of the correlation between resistance range and fitness (inclusion of plasmid size as a term in linear regression of fitness cost against resistance range: *t* = 0.003, *P* = 0.997). It is important to note that the number of phenotypic resistances a plasmid carries is not synonymous with the number of resistance genes a plasmid encodes. Many resistances require multiple genes to work, while many plasmids carry multiple independent resistance genes for one antimicrobial. As data on the number of resistance genes carried by a plasmid is scarcer, we were unable to include this information in the analysis.

### Chromosomal genes contribute to the cost of plasmids

**Figure 6:**
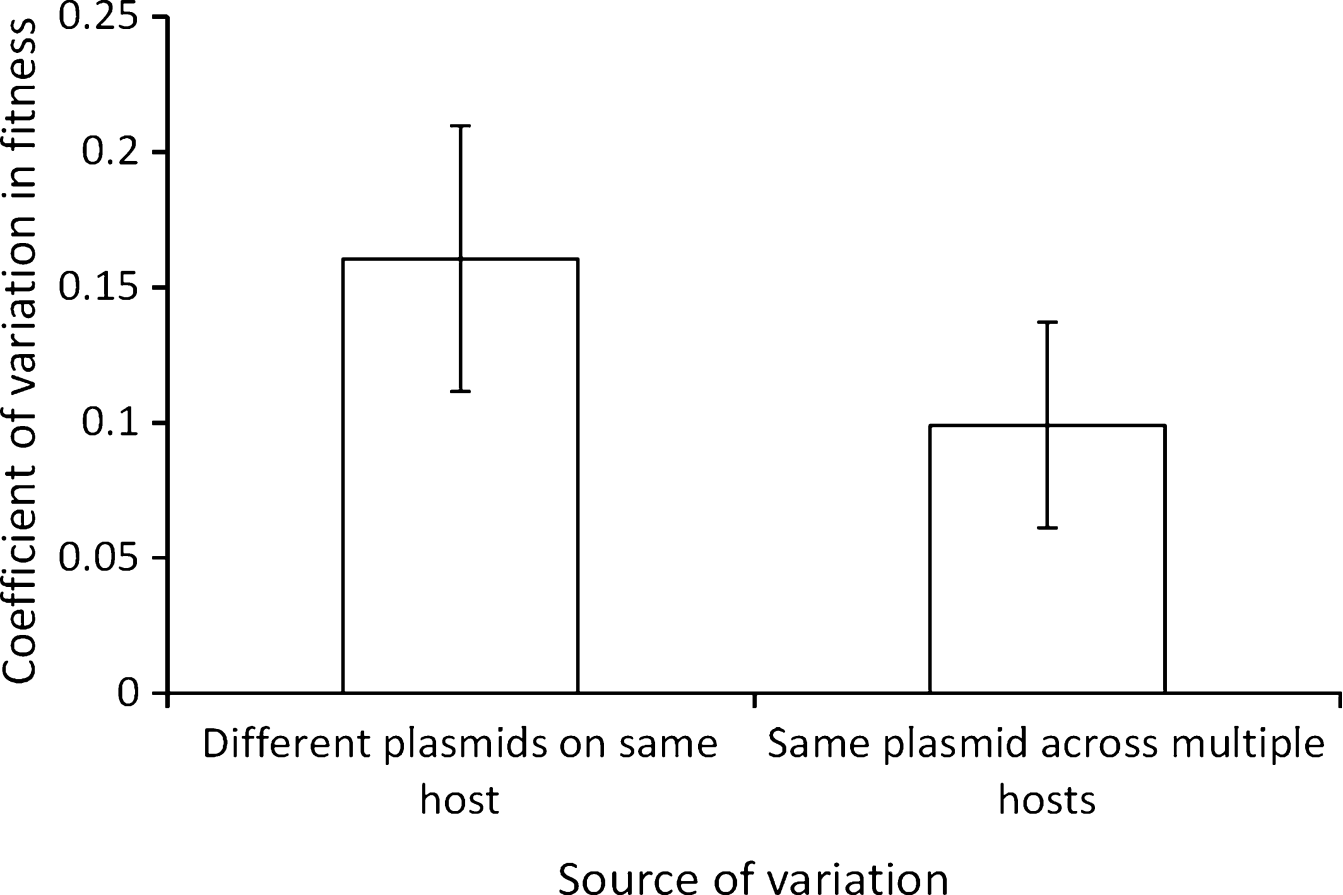
Both plasmid factors and bacterial factors contribute the size of the cost of acquiring a plasmid. Bars show the mean coefficient of variation (± SEM) for papers which either measured the cost of several different plasmids on a single host, or measured the cost of a single plasmid in different bacterial hosts.

In several papers (*n* = 5), the cost of a plasmid was assayed in multiple different bacterial hosts, while in several other papers (*n* = 6), the costs of several different plasmids have been assayed on a single host. It is striking that the variance for the same plasmid on different naïve hosts often appears to be as large as the variation between different plasmids on one host. If the same plasmid has different costs in different hosts, it implies that the cost of a plasmid is caused as much by properties of the bacteria as by any property the plasmid itself. Ideally, factorial designs would be used, where the costs of different plasmids are assayed across the same set of host strains, to simultaneously assess the importance of host and plasmid properties in determining the cost of plasmid acquisition. This would put the proportion of variance due to host properties in the context of a relevant biological comparison. However, from the existing published data, the mean coefficient of variation in fitness when a plasmid is assayed across multiple hosts is 9.8%. Interesting, this is not significantly different (Fig.6; one-way anova: *F*_1,9_ = 0.917, *P* = 0.363) from the mean coefficient of variation in fitness when multiple plasmids are assayed on one host (16%). This suggests that host genes or traits are at least as important as any plasmid gene or trait in determining the fitness cost of a plasmid.

### Biochemical mechanisms of chromosomal resistance mutations

Chromosomal mutations which result in resistance can do so by a variety of mechanisms, which can broadly be divided into target-site and non-target-site mechanisms (Andersson and Hughes 2010). The former of these arises due to mutations in the gene encoding the protein to which the antibiotic physically binds. The latter group is far more diverse. Common mechanisms include up-regulation of so-called defence genes, such as efflux pumps and enzymes that degrade or modify antibiotics, for which we identified six papers (Lindgren et al. 2005; Marcusson et al. 2009; Abdelraouf et al. 2011; Guo et al. 2012; Kunz et al. 2012; Olivares et al. 2012) and two papers (Marciano et al. 2007; Moya et al. 2008), respectively. We also identified several papers where resistance was due to reduced expression or loss of function mutations. These include reduced expression of porin genes (Abdelraouf et al. 2011), loss of reducing enzymes (Sandegren et al. 2008), and loss of intracellular transporters (Pränting et al. 2008; Castaneda-Garcia et al. 2009). We also identified so-called by-pass resistance to the peptide deformylase actinonin (Paulander et al. 2007; Zorzet et al. 2012), where resistance evolves via bypassing the need for the inhibited enzyme, as well as resistance to lysostaphin via alterations to cell-wall structure (Kusuma et al. 2007). Finally, we identified several papers reporting the cost of resistance due to small colony variants (SCVs) (Norström et al. 2007; Seaman et al. 2007; Pränting and Andersson 2011). This is where a mutation markedly reduces the rate of a cell's respiration, and consequently results in resistance to a wide diversity of antimicrobials, heavy metals, and other stresses.

**Figure 7:**
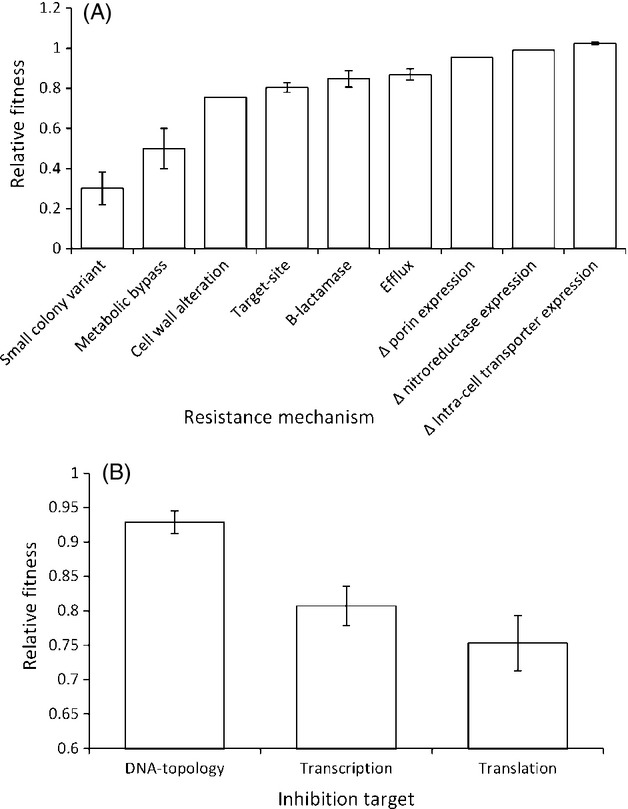
(A): The mechanistic basis of chromosomal resistance significantly affects the cost of resistance. Bars represent mean fitness (± SEM, if at least 2 published reports exist). 7(B): the metabolic pathway affected by target-site resistance significantly affects the cost of resistance. Bars represent the mean fitness cost of a single resistance mutation to either a DNA-topoisomerase inhibitor, a transcription inhibitor, or a translation inhibitor.

Overall, we find that the molecular basis of chromosomal resistance mutations significantly affects the cost of resistance (Fig.7A; one-way anova on all mechanisms with at least two data points: *F*_5,67_ = 7.41, *P* < 0.001). However, given the diversity of mechanisms and the number of replicates of each mechanism, it is unsurprising that post hoc analysis does not add much additional detail. Specifically, SCVs are found to be more costly than all of the other mechanisms of resistance (Bonfferoni corrected *t*-tests, *P* < 0.05 in all cases), while reduced intracellular transport is also significantly less costly than by-pass resistance to actinonin. This lack of significant variation may reflect the actual biology but may also be due the scale of our analysis and consequently the many other factors which will co-vary with resistance mechanism.

Target-site resistance can be additionally divided based on the biological process targeted by antibiotics. Broadly this encompasses inhibitors of DNA-topoisomerases (fluoroquinolones, *n* = 11 papers), RNA-polymerase inhibitors (rifampicin and myxopyronin *n* = 17 papers), translation inhibitors (aminoglycosides, macrolides, linezolid, mupirocin, and fusidic acid (*n* = 29) and cell-wall synthesis inhibitors (beta-lactams, *n* = 1, not included in statistics). The cost of resistance varied significantly between the different biochemical pathways being inhibited (Fig.7B; one-way anova: *F*_2,54_ = 4.49, *P* < 0.05). Specifically, mutations in DNA-topoisomerases were less costly than mutations effecting translation (post hoc Tukey test, mean difference = 0.180, *P* < 0.05). Mutations in RNA-polymerase genes were not significantly different from either mutations affecting translation (post hoc Tukey test, mean difference = 0.054, *P* = 0.908), or mutations affecting DNA-topoisomerases (post hoc Tukey test, mean difference = 0.126, *P* = 0.181). It is important to note that these results are for single mutations, and therefore, it cannot be inferred that clinically evolved resistance will necessarily reflect this pattern. Clinical isolates often possess multiple resistance mutations, but the exact number varies between different antimicrobials. For example, fluoroquinolone-resistant isolates normally possess multiple mutations (e.g. Jalal et al. 2000; Wang et al. 2001; Weigel et al. 2001), while in contrast rifampicin resistance is normally due to a single mutation in an RNA-polymerase gene (e.g. Wehrli 1983; Somoskovi et al. 2001).

### The role of epistasis

The evolution of high levels of resistance to an antibiotic via mutation sometimes involves the substitution of multiple mutations. For example, the majority fluoroquinone-resistant clinical isolates harbour multiple mutations in DNA-topoisomerases and often carry mutations in efflux pump repressors (e.g. Jalal et al. 2000; Wang et al. 2001; Weigel et al. 2001). If interactions between resistance mutations are additive, then the fitness of a strain carrying multiple resistance mutations is equal to the product of the fitness of strains carrying each mutation individually (reviewed in de Visser et al. 2011). For example, the fitness of a strain carrying mutations A and B, *w*_*AB*_, will be equal to *w*_Ab_* w_*bA*_. In this scenario, the cost of resistance will increase linearly with the number of resistance mutations acquired. Alternatively, it is possible that epistasic interactions between resistance mutations shape the cost of resistance. If positive epistasis occurs between resistance mutations, the fitness of strains carrying multiple mutations will be greater than expected from an additive model, that is, *w*_*AB*_> *w*_Ab_* w_*bA*_. Positive epistasis will promote the evolution of resistance, because successive resistance mutations will incur diminishing costs; if epistasis is strongly positive, it is even conceivable that strains carrying multiple mutations that are individually costly may even pay no cost for resistance. Under negative epistasis, the fitness of strains carrying multiple resistance mutations is less than what would be expected under an additive model, that is, *w*_*AB*_< *w*_Ab_* w_*bA*_. Negative epistasis will prevent the evolution of resistance, because successive resistance mutations will aggravate each other's' costs.

**Figure 8:**
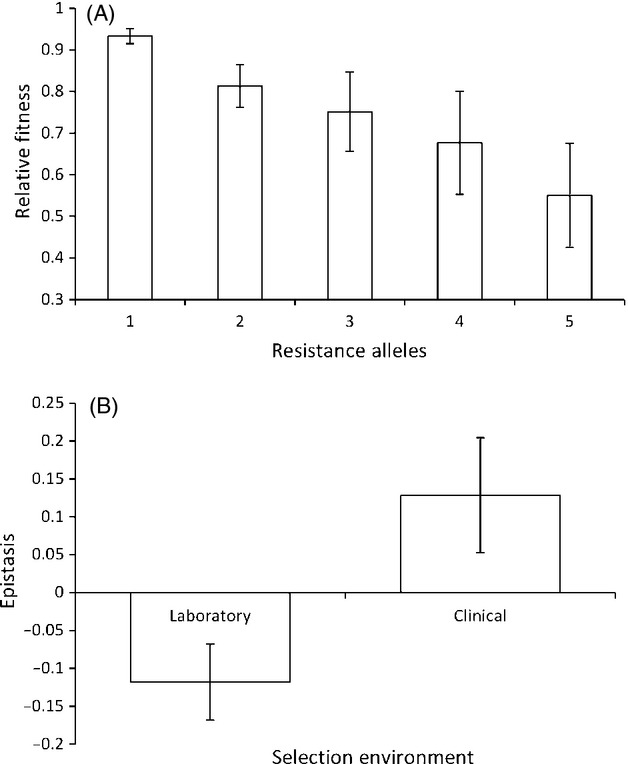
(A): The cost of stepwise resistance increases with each successive step. Bars show mean relative fitness (±SEM) from 13 papers which measured the stepwise evolution of resistance. 7 of these papers only compared strains with 1 or 2 resistance alleles or mutations. The remaining 6 then went on to study a third level of resistance, and 3 of these also reported the costs of acquiring a fourth and fifth resistance mutation. 8(B): There is significant negative epistasis between laboratory evolved alleles, but no significant epistasis between clinically evolved alleles. Bars represent the mean difference between no epistasis (where the cost of a second mutation is the same as the first), and what is actually observed from the 13 papers. A negative value indicates a greater than expected cost, and a positive result a smaller than expected cost.

We identified 13 papers which had analysed the stepwise evolution of resistance. Although some of these papers had compared stepwise resistant isolates with up 5 mutations, due to progressively smaller sample sizes, our analysis will focus on strains with just one and two mutations. Overall, strains harbouring two resistance mutations are significantly less fit than strains with one resistance mutation (Fig.8A; paired *t*-test: *t* = 2.47, df = 12, *P* < 0.05). This is a key result, because it implies that there is an overall cost to increasing antibiotic resistance. To test for epistasis, for each paper, we calculated the expected fitness of strains with 2 mutations if both mutations simply had the same effect as the mean first mutation (i.e. taking the square of the fitness cost of first-order resistant strains). Subtracting this expected fitness from the observed fitness of second-order mutants reveals no significant epistasis (one-sample *t*-test: *t* = 1.21, df = 12, *P* = 0.250). However, if we ignore the direction of epistasis by using the absolute of the difference between predicted and observed fitness, we find that the fitness costs of the second mutation to fix is significantly nonrandom (one-sample *t*-test: *t* = 3.32, df = 12, *P* < 0.01). In other words, the first test failed to detect epistasis because some papers found significant negative epistasis, while others found significant positive epistasis.

These 13 papers have used a variety of different methods to generate the stepwise increases in resistance. Ten of these papers used constructs of independently evolved laboratory mutations, while the remaining three papers used combinations of alleles which had evolved in clinical environments which were then transferred onto isogenic backgrounds for further analysis. The key difference between these methods is that the lab studies examine interactions between artificially generated combinations of mutations, whereas clinical studies examine interactions between mutations that were naturally assembled into combinations. Interestingly, we find no significant epistasis for these three papers which utilized clinical alleles (one-sample *t*-test: *t* = 1.69, df = 2, *P* = 0.232), although the sample size here is very small. In contrast, for the 10 papers using laboratory alleles the analysis reveals significant negative epistasis (one-sample *t*-test: *t* = 2.36, df = 9, *P* < 0.05), and therefore laboratory evolved second-order resistance mutations have significantly higher costs than predicted from the effects of the first-order mutations. There is also a significant difference in epistasis between the two different sources of alleles (Fig.8B; one-way anova, clinical versus laboratory, *F*_1,11_ = 5.93, *P* < 0.05).

## Discussion

One of the most important challenges in understanding of antimicrobial resistance is to determine whether there are any broad, general features which can be applied across biological systems (Martinez et al. 2007; zur Wiesch et al. 2011). A considerable amount of work has been performed to determine the genetic and biochemical basis of antimicrobial resistance. Here, we attempted to determine whether the molecular basis of antimicrobial resistance can be connected to fitness at a broad scale. We find that the genetic basis of resistance plays a key role in determining its costs; specifically, we find that evolving resistance by plasmid acquisition tends to carry a much smaller cost than evolving resistance *de novo* by chromosomal mutation. In principle, the low cost of plasmid-associated resistance could help to explain why plasmids play such a predominant role in the evolution of resistance, particularly when combined with their ability to spread horizontally. In contrast, we found little evidence of systematic variation in fitness between alternative biochemical mechanisms of resistance. We are not arguing that the biochemical basis of resistance does not influence its costs; on the contrary, the biochemical effects of resistance can explain why some resistance mutations to the same antimicrobial are more costly than others (e.g. Andersson et al. 1986; Schrag and Perrot 1996; Macvanin et al. 2000; Zorzet et al. 2010). Perhaps, the diversity of resistance mutations to each antibiotic is responsible for the lack of explanatory power, although this could also be an artefact of the scale of our analysis.

### Why does plasmid-mediated resistance carry a small cost?

One of the most striking features of the evolution of antimicrobial resistance by chromosomal mutations is the high degree of conservation in the molecular basis of resistance. Resistance often evolves due to mutations in the same conserved sequences in different species (Alekshun and Levy 2007), and sometimes even due to the same substitutions. The implication of this is that the evolution of resistance by chromosomal mutation in a bacterial lineage represents a true *de novo* instance of resistance evolution. It is therefore unlikely that bacteria will initially possess adaptations to offset the cost of mutations in these genes, although it has been shown that bacteria can rapidly acquire such adaptations through compensatory evolution (reviewed in Andersson and Hughes 2010). In contrast, when bacteria evolve resistance by acquiring a plasmid they are obtaining a resistance determinant that has already experienced selection to minimize its cost in previous hosts. For example, it has been shown that plasmids genes are enriched for biosynthetically cheap amino acids relative to analogous chromosomal genes (Nogueira et al. 2009), demonstrating selection to minimize the cost of plasmid-encoded proteins. Additionally, bacteria can be ‘cured’ of plasmids by segregational loss during cell division. It is therefore possible that when bacteria evolve resistance by acquiring a plasmid, they are simply re-acquiring a plasmid that they previously carried during their evolutionary past. If this is the case, then bacterial chromosomes may already carry compensatory mutations that offset the cost of a newly acquired plasmid. In support of this argument, laboratory studies have shown that bacteria can rapidly adapt to plasmids carrying resistance genes, eliminating the cost of plasmid carriage. Crucially, this adaptation is as likely to be due to mutations on the host's chromosome as it is a mutation on the plasmid itself (Bouma and Lenski 1988; Dahlberg and Chao 2003; Dionisio et al. 2005). In some systems, this can result in hosts which harbour plasmids being fitter than their plasmid-free ancestors (Dionisio et al. 2005; Starikova et al. 2013). This host-plasmid coevolution could also explain why the cost of the same plasmid is so variable between different bacterial hosts – hosts in which a plasmid has a low fitness cost may have had a longer or more recent history of carrying that type of plasmid.

However, plasmid acquisition was still found to be costly, and this cost increased with increasing plasmid resistance range. In contrast, the size of a plasmid did not correlate with its fitness cost. As the number of total genes carried by a plasmid is correlated with its size, this is suggestive that resistance genes are more costly than the majority of plasmid-encoded traits. This could be due to resistance genes being relatively recent additions to the traits carried by plasmids; plasmids isolated prior to the antimicrobial-era do not carry antimicrobial resistance (Hughes and Datta 1983). Therefore, it is likely that the genes which encode resistance have had less time to adapt to being carried by plasmids than the majority of plasmid carried-traits.

### Epistasis and the evolution of resistance by mutation

Although plasmid acquisition provides bacteria with a potentially easy route to evolving resistance, spontaneous mutation is an important mechanism of resistance evolution. For example, mutation is the dominant mechanism of resistance evolution in the intracellular parasite *M. tuberculosis* (Sandgren et al. 2009; Muller et al. 2013) and in the opportunistic pathogen *P. aeruginosa* (Livermore 2002; Bonomo and Szabo 2006; Lister et al. 2009). The classic paradigm is that resistance evolution occurs by a few mutations of large effect. However, there is growing evidence that high levels of antibiotic resistance evolve by the substitution of multiple mutations that confer resistance to the same antibiotic (Weinreich et al. 2006; Bruchmann et al. 2013; Farhat et al. 2013; Zhang et al. 2013). When resistance evolves by mutations in multiple genes, epistatic interactions between resistance mutations have the potential to influence the overall cost associated with resistance. Previous work has shown that epistasis between mutations that confer resistance to alternative antibiotics is widespread, and there is an overall tendency towards positive epistasis (Trindade et al. 2009; Ward et al. 2009). We find that epistasis is also widespread between pairs of mutations that confer resistance to the same antibiotic, which is in agreement with previous work on the genetics of rifampicin resistance (Hall and MacLean 2011). Our analysis makes the intriguing suggestion that selection in clinical environments leads to the evolution of combinations of resistance mutations that pay a minimal epistatic cost of resistance. This analysis also suggests that there is considerable scope for exploring the role of epistasis in resistance evolution in clinical environments.

### Biases in the cost of resistance literature

The cost of resistance has now been measured in well over 100 studies, and this literature provides an important resource for understanding the evolution of antibiotic resistance. However, it is important to point out that there are some important biases in the cost of resistance literature. Perhaps most importantly, this reviewed focussed on *in-vitro* measures of fitness cost, for which a considerable amount is known. There is considerably less work on the evolutionary dynamics of resistance *in-vivo,* such as in patients who have been treated with antibiotics or in environments that are involved in pathogen transmission (but see Gustafsson et al. 2003). There is also a bias in the literature towards working with antibiotics that are experimentally tractable, rather than clinically relevant. For example, *β*-lactams are currently the most commonly used antimicrobials (Goossens et al. 2007; Adriaenssens et al. 2011). However, there is considerably more known about the fitness costs of rifampicin and aminoglycoside resistance, both of which are less commonly used. This is not say research about rifampicin and aminoglycoside resistance is not important and interesting. However, it is noticeable that the costs associated with these mechanisms of resistance are far better characterized than the costs of β-lactam resistance. Lastly, estimates of the cost of resistance associated with mobile genetic elements are lacking. Resistance genes are horizontally transferred by a wide diversity of mobile genetic elements, including plasmids, transposons, bacteriophages, genomic islands, integrons, and ICEs (reviewed in Barlow 2009). However, only costs associated with resistance carrying plasmids have been studied in any detail. Notable exceptions to this include the four transposons included in our analysis (Enne et al. 2005; Foucault et al. 2010; Starikova et al. 2012), as well as several papers published since our search was performed (e.g. Knight et al. 2013; Starikova et al. 2013). There are also costs of MGE acquisitions where the genetic background was not controlled (e.g. Foucault et al. 2009; Corich et al. 2010). However, in general, this is an area where considerable extra research is required.

## Data archiving statement

Data for this study are available from the Dryad Digital Repository: http://doi.org/10.5061/dryad.p745m.

## Supporting Information

**Appendix S1.** Publications included in meta-analysis.
