# Supplementary material for: The genetic basis of the fitness costs of antimicrobial resistance: a meta-analysis approach
Source: Evol Appl. 2014 Dec 12;8(3):284–95. doi: 10.1111/eva.12202 (PMC4380922; doi:10.1111/eva.12202)
Supplement: Supplementary file 1 — Appendix S1. Publications included in meta-analysis. [file eva0008-0284-sd1.docx]

**Appendix: Publications included in meta-analysis.**

Abdelraouf, K., S. Kabbara, et al. (2011). ‘Effect of multidrug resistance-conferring mutations on the fitness and virulence of Pseudomonas aeruginosa’. Journal of Antimicrobial Chemotherapy **66**(6): 1311-1317.

Albarracín Orio, A. G., G. E. Piñas, et al. (2011). ‘Compensatory Evolution of <italic>pbp</italic> Mutations Restores the Fitness Cost Imposed by β-Lactam Resistance in <italic>Streptococcus pneumoniae</italic>’. PLoS Pathog **7**(2): e1002000.

Almofti, Y. A., M. Dai, et al. (2011). ‘The physiologic and phenotypic alterations due to macrolide exposure in Campylobacter jejuni’. Int J Food Microbiol **151**(1): 52-61.

Balsalobre, L., M. J. Ferrándiz, et al. (2011). ‘Nonoptimal DNA Topoisomerases Allow Maintenance of Supercoiling Levels and Improve Fitness of Streptococcus pneumoniae’. Antimicrobial Agents and Chemotherapy **55**(3): 1097-1105.

Barrick, J. E., M. R. Kauth, et al. (2010). ‘Escherichia coli rpoB Mutants Have Increased Evolvability in Proportion to Their Fitness Defects’. Molecular Biology and Evolution **27**(6): 1338-1347.

Besier, S., A. Ludwig, et al. (2008). ‘Linezolid resistance in Staphylococcus aureus: Gene dosage effect, stability, fitness costs, and cross-resistances’. Antimicrobial Agents and Chemotherapy **52**(4): 1570-1572.

Billington, O. J., T. D. McHugh, et al. (1999). ‘Physiological cost of rifampin resistance induced in vitro in Mycobacterium tuberculosis’. Antimicrob Agents Chemother **43**(8): 1866-1869.

Binet, R. and A. T. Maurelli (2005). ‘Fitness Cost Due to Mutations in the 16S rRNA Associated with Spectinomycin Resistance in Chlamydia psittaci 6BC’. Antimicrobial Agents and Chemotherapy **49**(11): 4455-4464.

Binet, R. and A. T. Maurelli (2007). ‘Frequency of Development and Associated Physiological Cost of Azithromycin Resistance in Chlamydia psittaci 6BC and C. trachomatis L2’. Antimicrobial Agents and Chemotherapy **51**(12): 4267-4275.

Björkman, J., D. Hughes, et al. (1998). ‘Virulence of antibiotic-resistant Salmonella typhimurium’. Proceedings of the National Academy of Sciences **95**(7): 3949-3953.

Björkman, J., I. Nagaev, et al. (2000). ‘Effects of Environment on Compensatory Mutations to Ameliorate Costs of Antibiotic Resistance’. Science **287**(5457): 1479-1482.

Castaneda-Garcia, A., A. Rodriguez-Rojas, et al. (2009). ‘The Glycerol-3-Phosphate Permease GlpT Is the Only Fosfomycin Transporter in Pseudomonas aeruginosa’. Journal of Bacteriology **191**(22): 6968-6974.

Cohan, F. M., E. C. King, et al. (1994). ‘Amelioration of the Deleterious Pleiotropic Effects of an Adaptive Mutation in Bacillus-Subtilis’. Evolution **48**(1): 81-95.

Cottell, J. L., M. A. Webber, et al. (2012). ‘Persistence of Transferable Extended-Spectrum-β-Lactamase Resistance in the Absence of Antibiotic Pressure’. Antimicrobial Agents and Chemotherapy **56**(9): 4703-4706.

Criswell, D., V. L. Tobiason, et al. (2006). ‘Mutations conferring aminoglycoside and spectinomycin resistance in Borrelia burgdorferi’. Antimicrob Agents Chemother **50**(2): 445-452.

Dahlberg, C. and L. Chao (2003). ‘Amelioration of the Cost of Conjugative Plasmid Carriage in Eschericha coli K12’. Genetics **165**(4): 1641-1649.

De Gelder, L., J. M. Ponciano, et al. (2007). ‘Stability of a promiscuous plasmid in different hosts: no guarantee for a long-term relationship’. Microbiology **153**(2): 452-463.

Dionisio, F., I. C. Conceição, et al. (2005). ‘The evolution of a conjugative plasmid and its ability to increase bacterial fitness’. Biology Letters **1**(2): 250-252.

Ender, M., N. McCallum, et al. (2004). ‘Fitness Cost of SCCmec and Methicillin Resistance Levels in Staphylococcus aureus’. Antimicrobial Agents and Chemotherapy **48**(6): 2295-2297.

Enne, V. I., P. M. Bennett, et al. (2004). ‘Enhancement of host fitness by the sul2-coding plasmid p9123 in the absence of selective pressure’. Journal of Antimicrobial Chemotherapy **53**(6): 958-963.

Enne, V. I., A. A. Delsol, et al. (2005). ‘Assessment of the fitness impacts on Escherichia coli of acquisition of antibiotic resistance genes encoded by different types of genetic element’. Journal of Antimicrobial Chemotherapy **56**(3): 544-551.

Enne, V. I., A. A. Delsol, et al. (2004). ‘Rifampicin resistance and its fitness cost in Enterococcus faecium’. Journal of Antimicrobial Chemotherapy **53**(2): 203-207.

Foucault, M.-L., F. Depardieu, et al. (2010). ‘Inducible expression eliminates the fitness cost of vancomycin resistance in enterococci’. Proceedings of the National Academy of Sciences **107**(39): 16964-16969.

Gillespie, S. H., O. J. Billington, et al. (2002). ‘Multiple drug-resistant Mycobacterium tuberculosis: Evidence for changing fitness following passage through human hosts’. Microbial Drug Resistance **8**(4): 273-279.

Giraud, E., A. Cloeckaert, et al. (2003). ‘Fitness cost of fluoroquinolone resistance in Salmonella enterica serovar Typhimurium’. Journal of Medical Microbiology **52**(8): 697-703.

Guo, B. N., K. Abdelraouf, et al. (2012). ‘Predicting bacterial fitness cost associated with drug resistance’. Journal of Antimicrobial Chemotherapy **67**(4): 928-932.

Gustafsson, I., O. Cars, et al. (2003). ‘Fitness of antibiotic resistant Staphylococcus epidermidis assessed by competition on the skin of human volunteers’. Journal of Antimicrobial Chemotherapy **52**(2): 258-263.

Haenni, M. and P. Moreillon (2008). ‘Fitness Cost and Impaired Survival in Penicillin-Resistant Streptococcus gordonii Isolates Selected in the Laboratory’. Antimicrobial Agents and Chemotherapy **52**(1): 337-339.

Han, F., S. Pu, et al. (2009). ‘Fitness cost of macrolide resistance in Campylobacter jejuni’. International Journal of Antimicrobial Agents **34**(5): 462-466.

Hao, H., M. Dai, et al. (2009). ‘23S rRNA Mutation A2074C Conferring High-Level Macrolide Resistance and Fitness Cost in Campylobacter jejuni’. Microbial Drug Resistance **15**(4): 239-244.

Humphrey, B., N. Thomson, et al. (2012). ‘Fitness of Escherichia coli strains carrying expressed and partially silent IncN and IncP1 plasmids’. BMC Microbiology **12**(1): 53.

Hurdle, J. G., A. J. O'Neill, et al. (2004). ‘Analysis of Mupirocin Resistance and Fitness in Staphylococcus aureus by Molecular Genetic and Structural Modeling Techniques’. Antimicrobial Agents and Chemotherapy **48**(11): 4366-4376.

Jin, D. J. and C. A. Gross (1989). ‘Characterization of the pleiotropic phenotypes of rifampin-resistant rpoB mutants of Escherichia coli’. Journal of Bacteriology **171**(9): 5229-5231.

Johnsen, P. J., G. S. Simonsen, et al. (2002). ‘Stability, persistence, and evolution of plasmid-encoded VanA glycopeptide resistance in enterococci in the absence of antibiotic selection in vitro and in gnotobiotic mice’. Microbial Drug Resistance-Mechanisms Epidemiology and Disease **8**(3): 161-170.

Kunz, A. N., A. A. Begum, et al. (2012). ‘Impact of Fluoroquinolone Resistance Mutations on Gonococcal Fitness and in vivo Selection for Compensatory Mutations’. Journal of Infectious Diseases.

Kusuma, C., A. Jadanova, et al. (2007). ‘Lysostaphin-Resistant Variants of Staphylococcus aureus Demonstrate Reduced Fitness In Vitro and In Vivo’. Antimicrobial Agents and Chemotherapy **51**(2): 475-482.

Lindgren, P. K., L. L. Marcusson, et al. (2005). ‘Biological Cost of Single and Multiple Norfloxacin Resistance Mutations in Escherichia coli Implicated in Urinary Tract Infections’. Antimicrobial Agents and Chemotherapy **49**(6): 2343-2351.

Little, R., J. Ryals, et al. (1983). ‘rpoB mutation in Escherichia coli alters control of ribosome synthesis by guanosine tetraphosphate’. Journal of Bacteriology **154**(2): 787-792.

Macvanin, M., U. Johanson, et al. (2000). ‘Fusidic acid-resistant EF-G perturbs the accumulation of ppGpp’. Molecular Microbiology **37**(1): 98-107.

Marciano, D. C., O. Y. Karkouti, et al. (2007). ‘A fitness cost associated with the antibiotic resistance enzyme SME-1 beta-lactamase’. Genetics **176**(4): 2381-2392.

Marcusson, L. L., N. Frimodt-Møller, et al. (2009). ‘Interplay in the Selection of Fluoroquinolone Resistance and Bacterial Fitness’. PLoS Pathog **5**(8): e1000541.

Mariam, D. H., Y. Mengistu, et al. (2004). ‘Effect of rpoB mutations conferring rifampin resistance on fitness of Mycobacterium tuberculosis’. Antimicrob Agents Chemother **48**(4): 1289-1294.

Michon, A., N. Allou, et al. (2011). ‘Plasmidic <italic>qnrA3</italic> Enhances <italic>Escherichia coli</italic> Fitness in Absence of Antibiotic Exposure’. PLoS One **6**(9): e24552.

Millan, A. S., S. Garcia-Cobos, et al. (2010). ‘Haemophilus influenzae Clinical Isolates with Plasmid pB1000 Bearing bla(ROB-1): Fitness Cost and Interspecies Dissemination’. Antimicrobial Agents and Chemotherapy **54**(4): 1506-1511.

Mongkolrattanothai, K., L. Pumfrey, et al. (2009). ‘Acquisition of High-Level Mupirocin Resistance and Its Fitness Cost among Methicillin-Resistant Staphylococcus aureus Strains with Low-Level Mupirocin Resistance’. Journal of Clinical Microbiology **47**(12): 4158-4160.

Moya, B., C. Juan, et al. (2008). ‘Benefit of Having Multiple ampD Genes for Acquiring β-Lactam Resistance without Losing Fitness and Virulence in Pseudomonas aeruginosa’. Antimicrobial Agents and Chemotherapy **52**(10): 3694-3700.

Nagaev, I., J. Björkman, et al. (2001). ‘Biological cost and compensatory evolution in fusidic acid-resistant Staphylococcus aureus’. Molecular Microbiology **40**(2): 433-439.

Nessar, R., J. M. Reyrat, et al. (2011). ‘Genetic analysis of new 16S rRNA mutations conferring aminoglycoside resistance in Mycobacterium abscessus’. Journal of Antimicrobial Chemotherapy **66**(8): 1719-1724.

Norström, T., J. Lannergård, et al. (2007). ‘Genetic and Phenotypic Identification of Fusidic Acid-Resistant Mutants with the Small-Colony-Variant Phenotype in Staphylococcus aureus’. Antimicrobial Agents and Chemotherapy **51**(12): 4438-4446.

O'Neill, A. J., T. Huovinen, et al. (2006). ‘Molecular Genetic and Structural Modeling Studies of Staphylococcus aureus RNA Polymerase and the Fitness of Rifampin Resistance Genotypes in Relation to Clinical Prevalence’. Antimicrobial Agents and Chemotherapy **50**(1): 298-309.

Olivares, J., C. Alvarez-Ortega, et al. (2012). ‘Overproduction of the multidrug efflux pump MexEF-OprN does not impair Pseudomonas aeruginosa fitness in competition tests, but produces specific changes in bacterial regulatory networks’. Environmental Microbiology **14**(8): 1968-1981.

Paulander, W., S. Maisnier-Patin, et al. (2007). ‘Multiple mechanisms to ameliorate the fitness burden of mupirocin resistance in Salmonella typhimurium’. Molecular Microbiology **64**(4): 1038-1048.

Paulander, W., S. Maisnier-Patin, et al. (2009). ‘The Fitness Cost of Streptomycin Resistance Depends on rpsL Mutation, Carbon Source and RpoS (σS)’. Genetics **183**(2): 539-546.

Paulander, W., A. Pennhag, et al. (2007). ‘Caenorhabditis elegans as a Model To Determine Fitness of Antibiotic-Resistant Salmonella enterica Serovar Typhimurium’. Antimicrobial Agents and Chemotherapy **51**(2): 766-769.

Petersen, A., F. M. Aarestrup, et al. (2009). ‘The in vitro fitness cost of antimicrobial resistance in Escherichia coli varies with the growth conditions’. FEMS Microbiol Lett **299**(1): 53-59.

Pfister, P., N. Corti, et al. (2005). ‘23S rRNA base pair 2057–2611 determines ketolide susceptibility and fitness cost of the macrolide resistance mutation 2058A→G’. Proceedings of the National Academy of Sciences of the United States of America **102**(14): 5180-5185.

Pränting, M. and D. I. Andersson (2011). ‘Escape from growth restriction in small colony variants of Salmonella typhimurium by gene amplification and mutation’. Molecular Microbiology **79**(2): 305-315.

Pränting, M., A. Negrea, et al. (2008). ‘Mechanism and Fitness Costs of PR-39 Resistance in Salmonella enterica Serovar Typhimurium LT2’. Antimicrobial Agents and Chemotherapy **52**(8): 2734-2741.

Reynolds, M. G. (2000). ‘Compensatory evolution in rifampin-resistant Escherichia coli’. Genetics **156**(4): 1471-1481.

Rozen, D. E., L. McGee, et al. (2007). ‘Fitness Costs of Fluoroquinolone Resistance in Streptococcus pneumoniae’. Antimicrobial Agents and Chemotherapy **51**(2): 412-416.

Sandegren, L., A. Lindqvist, et al. (2008). ‘Nitrofurantoin resistance mechanism and fitness cost in Escherichia coli’. Journal of Antimicrobial Chemotherapy **62**(3): 495-503.

Sandegren, L., M. Linkevicius, et al. (2012). ‘Transfer of an Escherichia coli ST131 multiresistance cassette has created a Klebsiella pneumoniae-specific plasmid associated with a major nosocomial outbreak’. J Antimicrob Chemother **67**(1): 74-83.

Sander, P., B. Springer, et al. (2002). ‘Fitness Cost of Chromosomal Drug Resistance-Conferring Mutations." Antimicrobial Agents and Chemotherapy **46**(5): 1204-1211.

Schrag, S. J., V. Perrot, et al. (1997). ‘Adaptation to the fitness costs of antibiotic resistance in Escherichia coli’. Proceedings of the Royal Society of London. Series B: Biological Sciences **264**(1386): 1287-1291.

Seaman, P. F., D. Ochs, et al. (2007). ‘Small-colony variants: a novel mechanism for triclosan resistance in methicillin-resistant Staphylococcus aureus’. Journal of Antimicrobial Chemotherapy **59**(1): 43-50.

Shcherbakov, D., R. Akbergenov, et al. (2010). ‘Directed mutagenesis of Mycobacterium smegmatis 16S rRNA to reconstruct the in vivo evolution of aminoglycoside resistance in Mycobacterium tuberculosis’. Molecular Microbiology **77**(4): 830-840.

Srivastava, A., D. Degen, et al. (2012). ‘Frequency, Spectrum, and Nonzero Fitness Costs of Resistance to Myxopyronin in Staphylococcus aureus’. Antimicrobial Agents and Chemotherapy **56**(12): 6250-6255.

Starikova, I., K. Harms, et al. (2012). ‘A Trade-off between the Fitness Cost of Functional Integrases and Long-term Stability of Integrons’. PLoS Pathog **8**(11): e1003043.

Subbiah, M., E. M. Top, et al. (2011). ‘Selection Pressure Required for Long-Term Persistence of blaCMY-2-Positive IncA/C Plasmids’. Applied and Environmental Microbiology **77**(13): 4486-4493.

Sun, S., A. Negrea, et al. (2009). ‘Genetic analysis of colistin resistance in Salmonella enterica serovar Typhimurium’. Antimicrob Agents Chemother **53**(6): 2298-2305.

Trindade, S., A. Sousa, et al. (2009). ‘Positive Epistasis Drives the Acquisition of Multidrug Resistance’. PLoS Genet **5**(7): e1000578.

Tubulekas, I. and D. Hughes (1993). ‘Suppression of rpsL phenotypes by tuf mutations reveals a unique relationship between translation elongation and growth rate’. Mol Microbiol **7**(2): 275-284.

Vickers, A. A., A. J. O'Neill, et al. (2007). ‘Emergence and maintenance of resistance to fluoroquinolones and coumarins in Staphylococcus aureus: predictions from in vitro studies’. Journal of Antimicrobial Chemotherapy **60**(2): 269-273.

Wichelhaus, T. A., A. Ludwig, et al. (2006). ‘Linezolid resistance in Staphylococcus aureus - gene dosage effect, stability, cross-resistance and fitness’. International Journal of Medical Microbiology **296**: 121-121.

Yates, C. M., D. J. Shaw, et al. (2006). ’Enhancement of bacterial competitive fitness by apramycin resistance plasmids from non-pathogenic Escherichia coli’. Biol Lett **2**(3): 463-465.

Zorzet, A., J. M. Andersen, et al. (2012). ‘Compensatory mutations in agrC partly restore fitness in vitro to peptide deformylase inhibitor-resistant Staphylococcus aureus.’ Journal of Antimicrobial Chemotherapy **67**(8): 1835-1842.

Zund, P. and G. Lebek (1980). ‘Generation time-prolonging R plasmids: correlation between increases in the generation time of Escherichia coli caused by R plasmids and their molecular size.’ Plasmid **3**(1): 65-69.
